# Cardiac Troponin, Cognitive Function, and Dementia: A Systematic Review

**DOI:** 10.14336/AD.2022.0818

**Published:** 2023-04-01

**Authors:** Michelle H Zonneveld, Denise Abbel, Saskia le Cessie, J. Wouter Jukema, Raymond Noordam, Stella Trompet

**Affiliations:** ^1^Department of Internal Medicine, Section of Gerontology and Geriatrics, Leiden University Medical Center, 2333 ZA Leiden, the Netherlands.; ^2^Department of Cardiology, Leiden University Medical Center, 2333 ZA Leiden, the Netherlands.; ^3^Department of Clinical Epidemiology, Leiden University Medical Center, 2333 ZA Leiden, the Netherlands.; ^4^Department of Biomedical Data Sciences, Leiden University Medical Center, 2333 ZA Leiden, the Netherlands.; ^5^Netherlands Heart Institute, Utrecht, the Netherlands

**Keywords:** troponin, cognitive function, dementia, cardiovascular disease, older adults

## Abstract

Elevated cardiac troponin, a biomarker of myocardial injury, has been found in individuals with brain damage and lower cognitive function. We conducted a systematic review to examine the association of troponin with cognitive function, incidence of dementia and dementia-related outcomes. PubMed, Web of Science and EMBASE were searched from inception to August 2022. Inclusion criteria were: (i) population-based cohort studies; (ii) troponin measured as determinant; and (iii) cognitive function in any metric or diagnosis of any type of dementia or dementia-related measures as outcomes. Fourteen studies were identified and included, with a combined total of 38,286 participants. Of these studies, four examined dementia-related outcomes, eight studies examined cognitive function, and two studies examined both dementia-related outcomes and cognitive function. Studies report higher troponin to be associated with higher prevalence of cognitive impairment (n=1), incident dementia (n=1), increased risk of dementia hospitalization (specifically due to vascular dementia) (n=1), but not with incident Alzheimer’s Disease (n=2). Majority of studies on cognitive function found elevated troponin also associated with worse global cognitive function (n=3), attention (n=2), reaction time (n=1) and visuomotor speed (n=1), both cross-sectionally and prospectively. Evidence regarding the association between higher troponin and memory, executive function, processing speed, language and visuospatial function was mixed. This was the first systematic review on the association between troponin, cognitive function, and dementia. Higher troponin is associated with subclinical cerebrovascular damage and might act as a risk-marker of cognitive vulnerability.

Troponin is a highly specific biomarker of myocardial injury. The protein is comprised of 3 subunits (T, C and I: from here onwards collectively referred to as troponin) and is expressed in striated muscle tissue such as the heart, and slow and fast skeletal muscle in order to contribute in excitation-contraction coupling (ECC) [1, 2]. Cardiac troponin is released into the circulation following cardiac myocyte cell death, often caused by acute athero-thrombotic plaque disruption due to coronary artery disease [3]. Although low concentrations (<14 ng/L) of troponin are considered normal, high levels of troponin (>14-30 ng/L) can be measured in the bloodstream 5 to 6 hours after acute myocardial infarction, with peak concentrations between 18 and 24 hours [4]. These concentrations can stay elevated up to 10 to 14 days. Sensitive automated assays enable the detection of circulating troponin, even at low concentrations in individuals with no clinically manifest myocardial damage or previously known cardiovascular disease (CVD) [5].

Elevated troponin levels have also been measured in patients following structural damage of the micro-vasculature in the brain, such as stroke [6], and in patients with the most severe white matter lesions [7], suggesting that (subclinical) damage to cerebral vasculature may trigger troponin release. Animal studies have also found troponin gene expression in the brain [8-10]. For example, transcriptomic analyses in mice found that the highest-ranked up-regulated gene, cardiac troponin C, codes for a calcium-binding protein that regulates actin binding in neurons [11]. Another study in mice identified the expression of a troponin gene (Tnnt1) in choroid plexus neuroepithelium and, although at lower concentrations, additionally found expression of this gene in the granule layer of the hippocampus [9]. Thus, troponin is not only present in cardiomyocytes but also expressed in the cerebral vasculature.

Increasing evidence suggests that higher plasma troponin levels are associated with accelerated cognitive decline and increased risk of dementia [10, 12-17]. CVD and cognitive impairment frequently co-exist in patients and share several risk-factors such as hypertension, diabetes mellitus and smoking [12]. Early identification of risk-markers of cognitive decline may provide an opportunity for the timely start of risk-reducing interventions. Hence, markers of (subclinical) cardiac dysfunction such as troponin may offer new potential, especially in those without clinical manifestation of a cardiac event [16]. Previous studies have pointed towards a relationship between troponin and cognitive changes, yet thus far this has not been reviewed systematically.

The aim of this study was to perform a systematic review of cohort studies that have investigated the association between serum troponin concentrations, cognitive function and dementia in older individuals.

## METHODS

The reporting of this systematic review was guided by the Preferred Reporting Items for Systematic reviews and Meta-Analyses (PRISMA) statement (see Supplement for checklist) [18]. This review is not registered.

### Search strategy

We performed a systematic literature search on PubMed, EMBASE and Web of Science, from inception to 12^th^ of August 2022. We included publications from population cohort studies that measured at least baseline troponin (C, I or T) and assessed cognitive function on a validated continuous cognitive function scale or diagnosis of any type of dementia or dementia-related outcomes. We did not restrict on age during our search as cognitive function is most often measured in older adults only. Details of our search strategy are presented in Supplementary material 1. Additional relevant articles were identified by studying the reference lists of the included studies.

### Eligibility criteria

Studies were eligible for inclusion if they were original studies and published in English. Studies were included if the determinant was serum troponin concentration and the outcome was at least one of the following: cognitive function in any metric, using clinically validated cognitive screening tests (e.g., the Mini-Mental Status Examination (MMSE)) and/or diagnosis of any type of dementia based on predefined diagnostic criteria (e.g., Diagnostic and Statistical Manual of Mental Disorders IV), or dementia-related outcomes (e.g., cognitive impairment). Quantitative studies of any design were eligible, including case-control, cohort or database registry, without restrictions on sample size. Studies were excluded if they were performed in children, non-English or if a full-text was unavailable. The following article types were excluded: (1) reviews; (2) systematic reviews; (3) comments; (4) editorials; (5) recommendations; (6) guidelines; (7) pilot studies; (8) letters to the editor; (9) author replies; (10) poster abstracts; (11) cost-effectiveness studies; (12) protocols; (13) case reports; (14) surveys.

### Study selection, data extraction, and risk of bias assessment

After completing the search, M.H.Z. performed the first round of title and abstract screening. The first round of screening aimed to exclude the irrelevant article types mentioned above. In the second round, two reviewers (M.H.Z. and D.A.) reviewed the content of the titles and abstracts of the 314 remaining identified articles independently according to the predefined inclusion and exclusion criteria. The selected articles were categorized into three groups: relevant; irrelevant; unsure. Articles categorized as irrelevant by both reviewers were excluded from the study without further consultation, and articles categorized as unsure were discussed with two additional independent researchers (S.T. and R.N.) until consensus was reached. Next, the full texts of the remaining articles were examined independently, and each reviewer made a list of articles to be included. The reviewers each excluded articles if the full texts were not in compliance with the inclusion and exclusion criteria (i.e., troponin was not the determinant, and cognitive function or diagnosis of any type of dementia or dementia-related outcomes were not the outcome). After lists were compared and consensus was reached, both researchers (M.H.Z. and D.A.) independently performed data extraction using a standard extraction form that gathered information regarding the study setting, participants, mean age, type of troponin measured, and outcomes including cognitive test scores, diagnosis of dementia (any type) or dementia related outcomes.

Two authors (M.H.Z. and D.A.) independently assessed the quality and risk of bias of using a modified Newcastle-Ottawa scale (NOS) for nonrandomized cohort studies (see Supplementary material 2) and discussed the assessment until consensus was reached. Studies were graded as having high (≥8 stars), moderate (5-7 stars), or low (0-4 stars) quality on the scale of 0 to 10 [19].

### Data synthesis

It was not possible to pool results using meta-analysis due to the relatively low number of studies included with similar outcome variables. However, we did harmonize the different study results in order to be able to compare outcomes per cognitive domain between the different studies in similar units (Fig. 2).

Cognitive domains include global cognitive function, memory, executive function, processing speed, attention, and language. From each study, the reported estimated beta (on a continuous scale and per natural log-unit increase troponin) from a linear regression, with 95% confidence interval and/or standard error, were extracted as raw data. The study by Schneider *et al* reported the difference in mean outcome per quintile of troponin, compared with a baseline category, which were used to estimate means and standard deviations per category, and beta by fitting a linear dose response model using the approach described in Crippa and Orsini [20]. As we had access to the original data from the PROSPER study (PROspective Study of Pravastatin in the Elderly at Risk) [21], we also recalculated the results from Wijsman *et al* (reported in tertiles of troponin) to harmonize and make consistent with other studies as seen in Figure 2. We kept the original results for reporting purposes in the results of the systematic review [12]. When results from multiple models were available, the results from the most fully adjusted models were used.

Next, we performed data harmonization. First, results from studies using test outcomes where a higher score indicated a worse performance, were inverted, such that a lower test score indicates a worse performance, which is similar to the measures of other cognitive domains. For continuous outcomes, the estimated regression co-efficients were transformed into standardized regression coefficients by dividing the estimated beta by the population standard deviation of the outcome. The standardized regression coefficients allow the comparison of effect sizes measuring the same cognitive domain, but using different scales. Corresponding 95% confidence intervals were calculated. Forest plots were designed using GraphPad Prism version 9.0.1 for Windows, GraphPad Software, San Diego, California, USA, www.graphpad.com.

**Figure 1.**
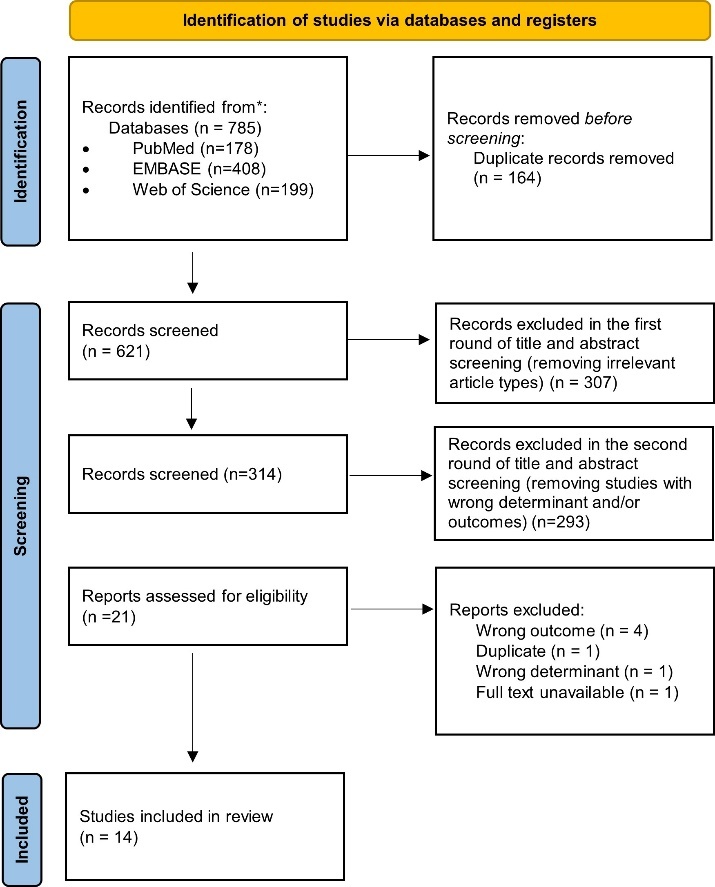
Flowchart of study selection.

## RESULTS

### Search and study characteristics

A total of 785 records were identified from the systematic search on PubMed (n=178), EMBASE (n=408) and Web of Science (n=199). After removing duplicates, 621 unique records remained. In total, 307 records were excluded in the first round of title and abstract screening on basis of article type, and an additional 293 articles were excluded in the second round using the formal inclusion and exclusion criteria. Following, the remaining twenty-one full-texts were assessed for eligibility (see Fig. 1), of which an additional 7 records were excluded. The characteristics of the fourteen remaining studies are reported in Table 1 (two studies examined both cognitive function as well as dementia-related outcomes). Eight studies were rated as high-quality, four studies as moderate and one study as low-quality (Supplementary material 3).

The mean age of study participants in the included studies ranged from 46.0 years to 86.0 years, with a sample size ranging between 88 and 9,472 participants. Eleven studies used troponin T as determinant, whereas three studies used troponin I. The majority of studies employed cross-sectional designs with some employing prospective analyzes. Overall, nine different cognitive domains and three different dementia-related outcomes were examined.

**Table 1:** Characteristics of the included studies

Dementia-related outcomes									
First author	Year	Study design	Sample size	Setting	Mean age in years	Follow-up in years	Type of troponin	Outcomes measured	Quality of study
Broersen* [17]	2020	Prospective	555	Prospective cohort with incident stroke Berlin	67.0	3	Cardiac troponin T	†Prevalence of cognitive impairment and troponin differences	High
Castro-Gomez [23]	2021	Cross-sectional	59	Memory and ALS (Amytrophic lateral sclerosis) unit patients and controls	72.1	-	Cardiac troponin T	†Difference in troponin between controls and Alzheimer patients	Moderate
Hilal [16]	2015	Case-control	158 cases, 35 controls	Memory clinic cases and controls	72.9 cases, 65.9 controls	-	Cardiac troponin T	†Cognitive impairment (yes/no) according to test battery	Moderate
Hasić [24]	2017	Cross-sectional	78	Hospice patients with and without dementia	81.0	-	Cardiac troponin I	†Difference in troponin between patients with and without dementia	Low
Tynkkynen [22]	2017	Prospective	7114	FINRISK - national population-based health survey	47.9	18	Cardiac troponin I	†Incident dementia ?Incident Alzheimer’s Disease using ICD codes	High
Schneider* [13]	2014	Prospective	9472	ARIC - Atherosclerosis Risk in Communities	62.5	13	Cardiac troponin T	†Incident dementia hospitalization using ICD codes	High
Cognitive function outcomes									
First author	Year	Study design	Sample size	Setting	Mean age in years		Type of troponin	Outcomes measured	Quality of study
Bertens [15]	2017	Cross-sectional & prospective	455	Leiden 85+ study	86.0		Cardiac troponin T	†Global cognition: MMSE	High
Broersen* [17]	2020	Cross-sectional & prospective	555	Prospective cohort with incident stroke Berlin	67.0		Cardiac troponin T	†Global cognition: MMSE, TICS-m	High
Choe [26]	2020	Cross-sectional	855	Biomarkers in Parkinson’s Disease study	66.9		Cardiac troponin I	†Global cognition: MoCA	Moderate
Gyanwali [25]	2021	Cross-sectional	434	Memory clinic patients	74.0		Cardiac troponin T	†Executive function: Verbal fluency test, color trails test 1 & 2 ?Attention: Digit span forward and backward test ?Language: 15-item modified Boston naming test ?Visuospatial function: Rey complex figure test-copy ?Visuomotor speed: Symbol digit modalities test ?Memory: Rey complex figure test-immediate and delayed recall and recognition, Hopkins verbal learning test-immediate and delayed test recall and recognition	High
Veugen [7]	2018	Cross-sectional	3011	The Maastricht Study	60.0		Cardiac troponin T	†Memory: Verbal-Learning test ?Processing speed: Stroop I & II test, Concept-Shifting test, Letter-Digit Substitution test ?Executive function/attention: Stroop III test	High
Van Vuren [28]	2019	Prospective	303	SABPA study - Sympathic activity and Ambulatory Blood Pressure in Africans	45.0		Cardiac troponin T	†Executive cognitive function: Stroop-Color-Word test	Moderate
Pokharel [10]	2019	Cross-sectional	9114	ARIC - Atherosclerosis Risk in Communities	63.4		Cardiac troponin T	†Verbal learning and memory: Delayed word recall test ?Executive function: Digit symbol substitution test ?Semantic fluency: Word fluency test	High
Von Rennenberg [27]	2022	Cross-sectional and prospective	1226	Berlin Aging Study II (BASE-II)	68.5		Cardiac troponin T	†Global cognition: Mini Mental State Examination ?Processing speed: Digit Symbol substitution test, Trail making test B ?Executive function: CERAD-Plus battery ?Memory: Items word list learning, word list recall, constitutional praxis recall, word list discriminability	High
Schneider* [13]	2014	Cross-sectional & prospective	9472	ARIC - Atherosclerosis Risk in Communities	62.5		Cardiac troponin T	†Verbal learning and recent memory: Delayed word recall test ?Executive function and processing speed: Digit symbol substitution test, Word fluency test	High
Wijsman [12]	2017	Cross-sectional & prospective	5407	PROSPER - PROspective Study of Pravastatin in the Elderly at Risk	75.3		Cardiac troponin T	†Executive function: Stroop III test ?Processing speed: Letter-digit coding test ?Memory: Picture-Word learning test immediate & delayed	High

Abbreviations: CERAD-Plus battery = Consortium to Establish a Registry for Alzheimer’s Disease battery; MMSE = Mini-Mental State Exam; TICS-m = Telephone Interview for Cognitive Status; MoCA = Montreal Cognitive Assessment; ICD = International Classification of Diseases. *Broersen and Schneider report both dementia-related as well as cognitive function outcomes and are therefore mentioned twice in this table.

### Troponin and the risk of dementia-related outcomes

Two studies reported differences in serum troponin between controls and dementia participants, and one study reported differences in serum troponin between controls and Alzheimer patients. Three studies reported the association between baseline troponin and incident dementia-related outcomes during follow up, of which one study assessed the incidence of cognitive impairment, two studies the incidence of dementia of which one specifically examined the incidence of Alzheimer’s Disease, and one study the risk of hospitalization due to dementia (Table 2)[13, 16, 22].

Schneider *et al.* showed that a baseline concentration of hs-cTnT was associated with an increased incidence of hospitalization due to dementia (ICD-9 code) (hs-cTnT ≥14 ng/L compared to hs-cTnT <3 ng/L, HR 2.68, 95% CI 1.87; 3.84) [13]. Similarly, Tynkkynen *et al.* found that elevated log hs-cTnI was associated with incident dementia (HR 1.12, 95% CI 1.02; 1.23), although this result was slightly attenuated after correcting for NT-proBNP (HR 1.10, 95% CI 0.99; 1.21) [22]. Broersen *et al*. reported a higher number of patients with cognitive impairment in the highest quartile of hs-cTnT (adjusted risk ratio 1.76, 95% CI 1.07; 2.90) [17]. Tynkkynen described a nonsignificant association between higher troponin and incident AD (according to ICD codes in hospital records, HR 1.06 per SD increase in troponin-I, 95% CI 0.95; 1.18) [22].

Hilal *et al.* reported baseline differences in hs-cTnT between controls, cognitive impairment no dementia (CIND) and dementia participants (p<0.001) [16]. Although they established a trend from CIND to dementia with higher tertiles of hs-cTnT, these associations were not significant. In line, Castro-Gomez did not find differences in serum troponin concentrations between controls and AD patients (data not reported) [23]. Likewise, Hasić *et al*. reported that there was no statistically significant difference in mean cTnI values between controls, AD group and vascular dementia group (p=0.74, no other values reported) [24], although the quality of this study was assessed as “low”.

**Figure 2.**
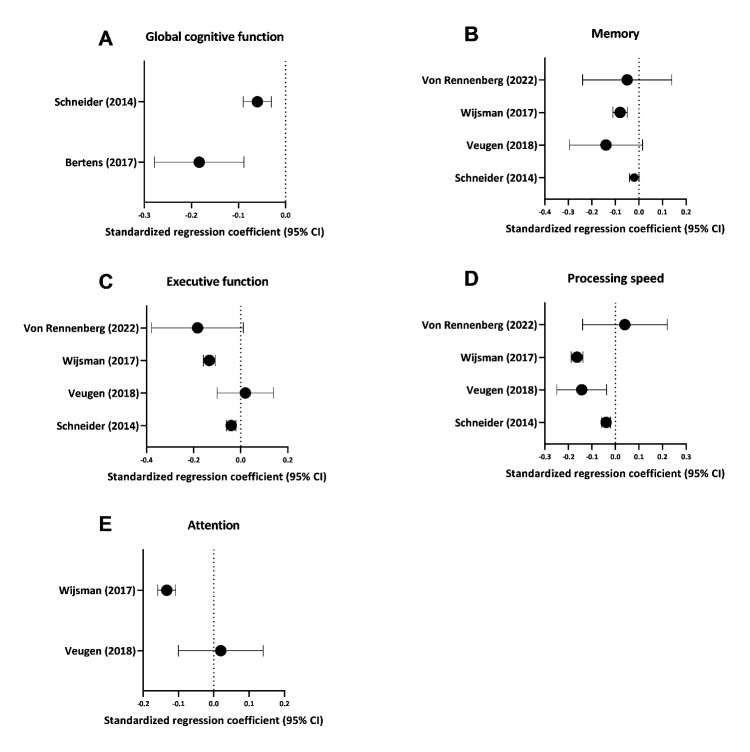
Cross-sectional associations of cardiac troponin T with cognitive function per domain. Data represent change in cognitive domain outcome (standardized regression coefficient, 95% confidence interval) per one log-unit increase of cardiac troponin T. Lower standardized mean difference indicates lower cognitive test performance.

### Troponin and cognitive function outcomes

#### Cross-sectional associations between baseline troponin and cognitive function

Seven studies reported on cross-sectional associations between serum troponin and cognitive function, of which five studies assessed global cognitive function, five studies assessed memory, four studies executive function, four studies processing speed, three studies attention, two studies language, one study reaction time, one study visuomotor speed, one study visuospatial function and one study visuo-construction (Table 2).

Three studies provided results demonstrating that higher troponin was associated with a lower global cognitive function [15, 17, 25]. For example, Bertens *et al.* showed that participants in the highest tertile of troponin had a 2.0-point lower baseline MMSE score compared to the lowest tertile (95% CI 0.73; 3.30) [15]. This is also confirmed by Gyanwali *et al*., whom found a -1.73 standard deviations (SD) lower (95% CI -2.70; -0.77) score at baseline on global cognition tests per unit increase in troponin [25]. On the other hand, Choe *et al.* described a nonsignificant association between lower MoCA test and higher hs-cTnI in Parkinson patients (beta -0.047 per ng/L increase in troponin, 95% CI -0.160; 0.066) after full covariable adjustments [26]. In line, Von Rennenberg did not find a significant association between baseline troponin and MMSE (data not reported in study) [27].

With regards to memory, studies presented mixed results. Three studies reported a lack of association, whereas two other studies reported higher serum troponin to be associated with lower scores on memory tests [7, 12, 13, 25, 27]. For example, Veugen *et al*. found higher troponin (cTnT) to be significantly associated with worse memory in participants ≥60 years of age (per 10-fold higher level of biomarker of cardiac injury, beta -0.31 SD, 95% CI -0.52; -0.11) [7].

**Table 2:** Results of the included studies.

Dementia-related outcomes		
First author	Year of publication	Results
Broersen* [17]	2020	†Prevalence of cognitive impairment at baseline was higher in patients in the highest quartile of hs-cTnT (adjusted risk ratio 1.76, 95% CI 1.07; 2.90).
Castro-Gomez [23]	2021	†There was no significant difference in serum troponin T between controls and Alzheimer’s Disease patients (data not reported).
Hilal [16]	2015	†Baseline differences in hs-cTnT were significant between the controls, cognitive impairment no dementia (CIND), and dementia participants (8.9 pg/mL (SD=6.5) *vs* 12.5 pg/mL (SD=9.4) *vs* 15.3 pg/mL (SD=9.9) respectively, p<0.001). ?A trend was found from CIND to dementia with higher tertiles of hs-cTnT, however these associations were not significant. For example, in dementia, an odds ratio of 4.13 (95% CI 0.83; 20.51) was found between highest tertile of hs-cTnT compared to the lowest tertile. ?Plasma hs-cTnT was associated with dementia and CIND, only when accompanied by presence of significant cerebrovascular disease.
Hasić [24]	2017	†There was no statistically significant difference in the mean cTnI values between the control group, Alzheimer’s Disease group and vascular dementia group (p=0.737, no further values reported).
Tynkkynen [22]	2017	†Log hs-cTnI was not associated with incident Alzheimer’s Disease during 18 years of follow-up (HR 1.06, 95% CI 0.95; 1.18). ?Log hs-cTnI was associated with incident dementia (HR 1.12, 95% CI 1.02; 1.23), although this association was annunciated when adjusting additionally for NT-proBNP (HR 1.10, 95% CI 0.99; 1.21).
Schneider* [13]	2014	†In prospective analyses, higher baseline concentrations of hs-cTnT were associated with an increased risk for dementia hospitalizations overall (hs-cTnT ≥14 ng/L compared to hs-cTnT <3 ng/L, HR 2.68, 95% CI 1.87; 3.84, p-trend<0.001). More specifically, higher troponin was associated with higher incident hospitalization for vascular dementia (P-trend = 0.029, HR not reported) but not for hospitalizations for AD (P-trend = 0.212, HR not reported).
Cognitive function outcomes		
First author	Year of publication	Results
Bertens [15]	2017	†Compared to participants in the lowest tertile of hs-cTnT and after full adjustments, participants in the highest tertile had a 2.0-point lower baseline MMSE score (95% CI 0.73; 3.3) and a steeper annual decline in MMSE during follow-up of 0.58 (95% CI 0.06; 1.1). ?A 1 log-unit increase of hs-cTnT was associated with 1.8 points (95% CI -2.7; -0.87) lower score on the MMSE after full adjustments. ?Higher hs-cTnT was also associated with faster annual decline on the MMSE (adjusted beta -0.62 per 1 log-unit increase of troponin, 95% CI -1.04; -0.20).
Broersen* [17]	2020	†Patients with elevated hs-cTnT scored lower on the baseline MMSE than patients without elevated hs-cTnT (median 27 (IQR 25-29) *vs* median 29 (IQR 27-30)). ?During follow-up, patients with elevated hs-cTnT scored lower on the TICS-m than patients with normal hs-cTnT (adjusted beta change in TICS-m score -1.42, 95% CI -2.33; -0.51).
Choe [26]	2020	†In a pooled cohort of Parkinson’s Disease patients at baseline, higher hs-cTnI was associated with a lower score on the MoCA (beta -0.173, 95% CI -0.300; -0.079). This association was not significant after full adjustments (per ng/L increase in troponin, beta -0.047 lower score on MoCA, 95% CI -0.160; 0.066).
Gyanwali [25]	2021	†At baseline, higher hs-cTnT (per 1 unit of 10-log increase) was associated with lower scores in global cognition (beta -1.73 SD, 95% CI -2.70; -0.77), executive function (beta -1.29 SD, 95% CI -2.09; -0.50), visuomotor speed (beta -1.05 SD, 95% CI -1.48; -0.62), attention (beta -1.01 SD, 95% CI -1.56; -0.46) and visuospatial function (beta -1.32 SD, 95% CI -2.13; -0.59). ?At baseline, higher hs-cTnT was not significantly associated with language (beta -1.85 SD, 95% CI -3.85; 0.14), and memory (beta -0.56 SD, 95% CI -1.20; 0.08) (reported significance p>0.05). ?During follow-up, hs-cTnT was associated with decline in global cognition, executive function and visuomotor speed (significant interaction between hs-cTnT and time for global cognition, p=0.001; executive function, p=0.012; and visuomotor speed, p=0.031).
Veugen [7]	2018	†In participants ≥60 years, higher cTnT was associated with lower memory (per log-ng/L higher troponin, beta -0.31 SD, 95% CI -0.52; -0.11, p<0.005), but not with information processing speed (beta -0.14 SD, 95% CI -0.30; 0.02, p=0.08) and with executive functioning and attention (beta 0.02 SD, 95% CI -0.16; 0.20, p=0.81). ?In participants ≥60 years of age, after full adjustments, higher cTnT was significantly associated with worse memory (beta -0.31 SD, 95% -0.52; -0.11, p<0.005) and more white matter lesions (p<0.01). This was not found in individuals <60 years of age. ?Associations between cTnT and cognitive performance domains became statistically significantly stronger with age; per 1-year older age, a -0.02 to -0.01 SD worse performance score on tests of cognitive domains per 10-fold higher level of cTnT.
Van Vuren [28]	2019	†In Black men, baseline Stroop color-word conflict test was inversely associated with %Δ cardiac troponin T (beta -0.32, 95% CI -0.53; -0.11, p≤0.01) during 3 years of follow-up. Thus, higher troponin was associated with a better performance on the Stroop test.
Pokharel [10]	2019	†Baseline hs-cTnT was not associated with 15-year cognitive change in any measure any measure (global Z-score, digit-symbol substitution test, delayed-word recall test, word fluency test). ?Participants with the highest levels of both hs-cTnT and NT-proBNP had excessive decline in global z scores versus participants with the lowest levels (beta -0.34, 95% CI -0.63; -0.04).
Von Rennenberg [27]	2022	†At baseline and during follow-up, there were no significant associations between hs-cTnT and MMSE (data not shown). ?After full adjustments, there were no significant associations between hs-cTnT and the following cognitive domains: processing speed (beta 0.04 SD, 95% CI -0.14; 0.22, p-value=0.682), executive function (beta -0.18 SD, 95% CI -0.38; 0.01, p-value=0.064), visuo-construction (beta 0.19 SD, 95% CI -0.01; 0.40, p-value=0.062), memory (beta -0.05 SD, 95% CI -0.24; 0.14, p-value=0.629) ?After full adjustments, per log higher hs-cTnT performance on the digit-symbol substitution test (processing speed) lowered by -2.68 points (95% CI -4.34; -1.02, p=0.002). ?During follow-up, higher log troponin was not associated with worse performance on the digit-symbol substitution test (beta -1.68 SD, 95% CI -3.54; 0.18, p=0.077). ?During follow-up, higher log troponin was associated with general cognitive decline (beta -0.59 SD, 95% CI -0.70; -0.47, p<0.001).
Schneider* [13]	2014	†In cross-sectional analyses, hs-cTnT ≥14 ng/L compared to hs-cTnT <3 ng/L was associated with lower scores on the digit-symbol substitution test (beta -1.83 SD, 95% CI -2.71; -0.96) and word-fluency test (beta -1.63 SD, 95% CI -2.64; -0.62), but not on the delayed-word recall test.
Wijsman [12]	2017	†Participants with higher hs-cTnT performed worse at baseline versus those with lower hs-cTnT on the Stroop test (70.09 seconds (SE=0.12) *versus* 65.91 seconds (SE=0.12), p<0.001), letter-digit coding test (22.28 digits coded (SE=0.30) *versus* 23.36 digits coded (SE=0.32), p<0.001), immediate picture-word learning test (9.28 pictures remembered (SE=0.08) *versus* 9.45 pictures remembered (SE=0.09), p=0.002) and delayed picture-word learning test (10.05 pictures remembered (SE=0.11) versus 10.31 pictures remembered (SE=0.12), p=0.001). ?Participants with higher hs-cTnT also showed steeper decline on all cognitive tests during 3 years of follow-up, independent of NT-proBNP.

Similar to memory, the association between higher troponin and executive function is ambiguous. One study reported per 1 unit of 10-log increase of troponin, an effect size of -1.29 SD lower score (95% CI -2.09; -0.50) on executive function tests [25], similar to results found by Schneider *et al.*, whilst Von Rennenberg (beta -0.18 SD, 95% CI -0.38; 0.01) and Veugen *et al.* reported no association (per log-ng/L higher troponin beta 0.02 SD, 95% CI -0.16; 0.20) [7, 27]. The findings concerning the association with processing speed are also inconclusive. Wijsman *et al.* found participants with higher hs-cTnT at baseline to perform worse on the Letter-Digit coding test (22.28 digits coded (SE=0.30) *versus* 23.36 digits coded (SE=0.32), p<0.001)[12], as did Schneider *et al.* (hs-cTnT ≥14 ng/L compared to hs-cTnT <3 ng/L, beta -1.83 SD, 95% CI -2.71; -0.96)[13], whereas Veugen *et al* reported per log-ng/L higher troponin, beta -0.14 SD (95% CI -0.30; 0.02), similar to Von Rennenberg (beta 0.04 SD, 95% CI -0.14; 0.22, p=0.682) [7].

Three studies reported on the association between troponin and attention. Wijsman *et al* and Gyanwali *et al* found higher hs-cTnT to be associated with lower scores [12, 25], whereas Veugen *et al* did not find this (per log-ng/L higher troponin, beta 0.02 SD, 95% CI -0.16; 0.20) [7]. Similarly, higher troponin was associated with lower language scores. Schneider *et al.* showed that a lower score on the Word-Fluency test (hs-cTnT ≥14 ng/L compared to hs-cTnT <3 ng/L, beta -1.63 SD, 95% CI -2.64; -0.62) was seen with higher hs-cTnT, a similar effect size with a larger confidence interval was found by Gyanwali *et al.* (per 1-unit of 10-log increase troponin, beta -1.85 SD, 95% CI -3.85; 0.14).

Last, Wijsman *et al*. found a slower selective attention to be associated with the highest tertile of troponin, compared to the lowest tertile (70.09 seconds (SE=0.12) *versus* 65.91 seconds (SE=0.12), p<0.001) [12]. Gyanwali *et al.* reports on the association between higher troponin with visuospatial function and visuomotor speed, where only the latter was significantly associated (beta -1.05 SD per 1 unit of 10-log increase in troponin, 95% CI -1.48; -0.62) [25]. Von Rennenberg did not find a significant association between higher troponin and visuo-construction (beta 0.19 SD, 95% CI -0.01; 0.40, p-value=0.062) [27].

#### Association between baseline troponin and annual change in cognitive function

Six studies reported prospective associations between higher troponin and cognitive function, of which five studies report on global cognitive function (n=5), two studies on memory (n=2), three studies processing speed (n=3), two studies executive function (n=2), one study language (n=1), one study attention (n=1), and one study reaction time (n=1) (Table 2).

Four studies found higher hs-cTnT to be associated with faster annual decline in global cognition [15, 17, 25]; for example, Bertens *et al.* found an annual decline of -0.62 (95% CI -1.04; -0.20) on the MMSE per log-unit increase in troponin, as well as Broersen and Gyanwali whom both reported faster global decline during follow-up (beta -1.42 points on TICS-m, 95% CI -2.33; -0.51) [15, 17, 25]. Von Rennenberg also report higher log troponin to be associated with general cognitive decline (beta -0.59 SD, 95% CI -0.70; -0.47, p<0.001) [27].

Pokharel did not find baseline hs-cTnT to be associated with 15-year cognitive change in global Z-score nor in executive function, language, memory and processing speed [10]. On the other hand, Wijsman *et al.* reported that participants with higher hs-cTnT showed significant steeper decline on both memory and processing speed tests during 3 years of follow-up, as well as with attention and reaction time [12]. Contrastingly, Van Vuren *et al* reported higher cardiac troponin T during follow-up to be associate with a better performance on the Stroop color-word conflict test (beta -0.32 seconds, 95% CI -0.53; -0.11) [28].

## DISCUSSION

This systematic review explored the associations of serum troponin with dementia-related outcomes and cognitive function in older adults. Studies reported associations between higher baseline troponin with prevalent cognitive impairment, incident dementia, higher risk for hospitalization due to vascular dementia, but not with incident Alzheimer’s Disease. Furthermore, most studies demonstrated elevated troponin was associated with worse global cognitive function, attention, reaction time and visuomotor speed, both cross-sectionally and prospectively.

Several mechanisms possibly explain the association between higher troponin, altered cognition and dementia-related outcomes. First, there is a large overlap in risk-factors contributing to cerebrovascular and cardiovascular disease. This may justify concurrent existence of cognitive dysfunction and increased concentrations of troponin. Even subclinical levels of troponin can be indicative of subclinical coronary calcification, a marker of subclinical atherosclerosis [29]. This may be a reflection of subclinical ischemia, which has previously also been associated with cognitive impairment [30]. Moreover, close correlations between severity of coronary atherosclerosis and troponin levels have been demonstrated using coronary angiography studies, potentially functioning as the source of both cognitive and cardiovascular malfunction [30, 31]. In line with this, inadequate left ventricular function can cause myocardial impairment and cerebral hypoperfusion which may also explain both elevated troponin and cognitive impairment [32].

Second, troponin has shown to be present in the smooth muscle vasculature of the brain. Raised serum concentrations of troponin may indicate early-stage cerebral microvasculature damage. This is further supported by studies who have found higher levels of hs-cTnT to be associated with (sub)clinical brain disease, including white matter hyperintensities and stroke, as well as studies who have found troponin gene expression in the visual cortex, choroid plexus neuroepithelium and hippocampus [8, 9, 33-35].

Third, excessive activation of adrenergic and renin-angiotensin systems may result as a consequence of (sub)clinical cerebrovascular and myocardial damage. Sympathoadrenal activation follows ischemic stroke, leading to myocardial changes such as myocytolysis and subsequent release of troponin and intracellular calcium [36, 37]. More specifically, stroke in tandem with insular cortex damage has been frequently associated with worse cardiac complications due to loss of autonomic control [38]. As a result, loss of autonomic control may lead to increased heart rate variability, blood pressure and QT prolongation, of which all 3 have previously been associated with lower cognitive performance [39-41]. Thus, troponin release due to activation of adrenergic and renin-angiotensin system may indirectly lead to cognitive changes. However, mutual causality potentially plays a role here, making it difficult to distinguish cause and effect.

Lastly, exaggerated immune response involving auto-antibodies and release of calcium may also be a contributing factor to take into consideration. As is now well-established, troponin is released following myocardial damage. A study in mice investigating long-term effects of troponin release found cardiac troponin-I to induce autoimmune inflammation of the myocardium [42]. Another study in mice showed administration of monoclonal antibodies to cTnI induced dilatation and cardiac dysfunction by augmenting voltage-dependent L-type Ca^2+^ currents [43]. Thus, cardiac dysfunction with consequences such as disrupted cerebral perfusion and cognitive impairment may result merely due to troponin release. However, additionally cardiac malfunction and inflammation can also cause the release of intracellular calcium as previously mentioned. The calcium ions that leaked following chronic stimulation can potentially cross the blood-brain barrier and lead to calcium overload. This can disrupt neuronal signaling which has been connected to hippocampal atrophy and Alzheimer’s disease [44]. Furthermore, calcium deposits in the brain have also been found in cases of frontal-subcortical dementia [44], and higher serum calcium levels previously been associated with faster cognitive decline [45].

In addition to the previously described mechanisms, it must also be noted that troponin has been linked to the development of other age-related diseases such as cancer and sarcopenia [46]. Of interest, malignancies have been previously inversely associated to the incidence of dementia [47], although in a limited sample and with likely limitations in the used study design. As cancer incidence is strongly dependent on age, perhaps the association between troponin C and dementia as described in the present systematic review is confounded by cancer. However, such information was generally not taken into account in the previously-performed studies. Similarly, troponin T3 has been associated with muscle weakness in sarcopenia, which in turn has also been associated with an increased risk of dementia [48]. Thus, longitudinal studies examining the association between various isoforms of troponin and the development of age-related diseases such as dementia and cancer are needed to further understand underlying biological pathways.

This is the first systematic review on troponin, cognitive function and dementia-related outcomes. We conducted a comprehensive literature review using multiple resources and included large number of outcomes and assessed the quality of the included studies. However, there are some limitations to this review that should be considered while interpreting the results. Due to the relatively low number of studies included we could not perform a formal meta-analysis as heterogeneity between studies could not be accounted for. Furthermore, we did not include the type of troponin as an inclusion/exclusion criterium. Although both troponin-T and I are markers of myocardial injury, actual mass concentrations differ largely, and assay results cannot be compared directly [49].

With regards to study population, only four of the fourteen included studies were (large) population-based cohorts (FINRISK (Tykkynen), Leiden 85+ study (Bertens), Berlin Aging Study II (Von Rennenberg) and Maastricht study (Veugen)), which increases the generalizability of our findings to the average population. However, the remaining 10 studies were populations with either significant history of cardiovascular disease such as atherosclerosis or older memory clinic patients, hampering the generalizability. Furthermore, the multitude of cognitive domains reduced comparability across studies, as outcomes differed strongly.

Despite these limitations, the results in this systematic review strongly proposes an interaction between cardiac troponin and the brain. The majority of the studies demonstrated significant associations between higher troponin, more dementia-related outcomes and lower cognitive function domains. Before clinical implications can be elucidated, it is important to further investigate causal relationships between troponin, cognitive impairment and perhaps subsequent dementia. The impact of troponin release and related repercussions could potentially enable earlier detection of subclinical cerebral dysfunction and allow development of novel diagnostic tools.

## Supplementary Materials

The Supplementary data can be found online at: www.aginganddisease.org/EN/10.14336/AD.2022.0818.
