# Supplementary material for: Cardiac Troponin, Cognitive Function, and Dementia: A Systematic Review
Source: Aging Dis. 2023 Apr 1;14(2):386–97. doi: 10.14336/AD.2022.0818 (PMC10017151; doi:10.14336/AD.2022.0818)
Supplement: Supplementary file 1 — The Supplementary data can be found online at: www.aginganddisease.org/EN/10.14336/AD.2022.0818. [file AD-14-2-386-s.pdf]

SUPPLEMENTARY DATA

Cardiac Troponin, Cognitive Function, and Dementia: A Systematic Review

Michelle H. Zonneveld[1,2], Denise Abbel[1], Saskia le Cessie[3,4], J. Wouter Jukema[2, 5], Raymond Noordam[1], Stella Trompet[1]

© 2022. Zonneveld MH et al. Published online at http://www.aginganddisease.org/EN/10.14336/AD.2022.0818

SUPPLEMENTARY DATA

Supplementary material 1.

Details of search strategy: Searched databases:

- PubMed: ("Troponin"[Mesh] OR "Troponin*"[tw] OR "hs-cTnT"[tw] OR "cTNT"[tw]) AND ("Dementia"[Mesh] OR "dementia"[tw] OR "Cognitive Dysfunction"[Mesh] OR "Cognitive"[tw] OR "Cognition"[Mesh] OR "Cognition"[tw] OR "CIND"[tiab] OR "executive function"[tw] OR "memory"[tw] OR "Memory"[Mesh] OR "processing speed"[tw] OR "pre-dementia"[tw] OR "predementia"[tw] OR "MCI"[tiab] OR "Alzheimer*"[tw])
    - 178 results
- Web of Science: TS=("Troponin*" OR "hs-cTnT" OR "cTNT") AND TS=("dementia" OR "Cognitive" OR "Cognition" OR "CIND" OR "executive function" OR "memory" OR "processing speed" OR "pre-dementia" OR "predementia" OR "MCI" OR "Alzheimer*")
    - 199 results
- EMBASE: (exp *troponin/ OR "Troponin*".ti. OR "hs-cTnT".ti. OR "cTNT".ti.) AND (exp dementia/ OR "dementia".mp. OR exp cognitive defect/ OR "Cognitive".mp. OR exp cognition/ OR "Cognition".mp. OR "CIND".ti,ab. OR "executive function".mp. OR "memory".mp. OR exp Memory/ OR "processing speed".mp. OR "pre-dementia".mp. OR "predementia".mp. OR "MCI".ti,ab. OR "Alzheimer*".mp.) NOT (conference OR conference abstract OR "conference review").pt.
    - 408 results

Supplementary material 2. Adapted Newcastle-Ottawa Scale

Newcastle-Ottawa quality assessment scale – adapted for <u>cross-sectional studies</u>

Selection (maximum 5 stars)

- <u>Representativeness of the exposed cohort</u>
    - Truly representative of the average in the target population * (all subjects or random sampling)
    - Somewhat representative of the average in the target population * (non-random sampling)
    - Selected group of users e.g., nurses, volunteers
    - No description of the derivation of the cohort
- <u>Selection of the non-exposed cohort</u>
    - Drawn from the same community as the exposed cohort *
    - Drawn from a different source
    - No description of the derivation of the non-exposed cohort
- <u>Ascertainment of exposure</u>
    - Validated measurement tool **
    - Non-validated measurement tool, but the tool is available or described*
    - No description of the measurement tool
- <u>Demonstration that outcome of interest was not present at start of study</u>
    - Yes *
    - No

Comparability (maximum 2 stars)

- <u>Comparability of cohorts on the basis of the design or analysis</u>
    - Study controls for age and sex *
    - Study controls for any additional factor *

Outcome (maximum 3 stars)

- <u>Assessment of the outcome:</u>
    - Independent blind assessment **
    - Record linkage **
    - Self-report
    - No description
- <u>Statistical test:</u>

© 2022. Zonneveld MH et al. Published online at http://www.aginganddisease.org/EN/10.14336/AD.2022.0818

  - The statistical test used to analyze the data is clearly described and appropriate, and the measurement of the association is presented, including confidence intervals and the probability level (p value) *
  - The statistical test is not appropriate, not described or incomplete

Newcastle-Ottawa quality assessment scale for case-control studies

Selection (maximum of 4 stars)

- Is the case definition adequate?
  - Yes, with independent validation *
  - Yes, e.g. record linkage or based on self-reports
  - No description
- Representativeness of the cases
  - Consecutive or obviously representative series of cases *
  - Potential for selection biases or not stated
- Selection of Controls
  - Community controls *
  - Hospital controls
  - No description
- Definition of Controls
  - No history of disease (endpoint) *
  - No description of source

Comparability (maximum of 2 stars)

- Comparability of cases and controls on the basis of the design or analysis
  - Study controls for age and sex *
  - Study controls for any additional factor *

Exposure (maximum of 3 stars)

- Ascertainment of exposure
  - Secure record (e.g. surgical records) *
  - Structured interview where blind to case/control status *
  - Interview not blinded to case/control status
  - Written self-report or medical record only
  - No description
- Same method of ascertainment for cases and controls
  - Yes *
  - No
- Non-Response rate
  - Same rate for both groups *
  - Non respondents described
  - Rate different and no designation

© 2022. Zonneveld MH et al. Published online at http://www.aginganddisease.org/EN/10.14336/AD.2022.0818

SUPPLEMENTARY DATA

Supplementary material 3. Quality assessment of studies using the modified Newcastle-Ottawa Quality assessment scale

Reference (First author and year)	Selection (maximum of 5 stars)				Comparability (maximum of 2 stars)	Outcome (maximum of 3 stars)			NOS score
	1	2	3	4	1	1	2		
Cross-sectional studies									
Bertens (2017)	*	*	**	N.A.	**	**	*		9/10
Broersen (2020)	*	*	**	N.A.	**	**	*		9/10
Castro-Gomez (2021)	*	-	**	*	-	**	*		7/10
Choe (2020)	-	-	**	N.A.	**	**	*		7/10
Gyanwali (2021)	*	*	**	N.A.	**	**	*		9/10
Hasić (2017)	-	*	**	N.A.	-	-	*		4/10
Veugen (2018)	*	*	**	N.A.	**	**	*		9/10
Van Vuren (2019)	-	*	**	N.A.	-	**	*		6/10
Tynkkynen (2017)	*	*	**	*	**	*	*		9/10
Pokharel (2019)	*	*	**	N.A.	**	**	*		9/10
Von Rennenberg (2022)	*	*	**	N.A.	**	**	*		9/10
Schneider (2014)	*	*	**	*	**	**	*		10/10
Wijsman (2017)	*	*	**	N.A.	**	**	*		9/10
**Reference (First author and year)**	**Selection (maximum of 4 stars)**				**Comparability (maximum of 2 stars)**	**Exposure (maximum of 3 stars)**			**NOS score**
	**1**	**2**	**3**	**4**	**1**	**1**	**2**	**3**	
Case-control studies									
Hilal (2015)	*	*	*	-	**	*	*	-	7/9

High (≥8 stars), moderate (5-7 stars), or low (0-4 stars).

Supplementary material . PRISMA 2020 Checklist

Section and Topic	Item #	Checklist item	Location where item is reported
**TITLE**			
Title	1	Identify the report as a systematic review.	Pg 1
**ABSTRACT**			
Abstract	2	See the PRISMA 2020 for Abstracts checklist.	Pg 2
**INTRODUCTION**			
Rationale	3	Describe the rationale for the review in the context of existing knowledge.	Pg 3
Objectives	4	Provide an explicit statement of the objective(s) or question(s) the review addresses.	Pg 3
**METHODS**			
Eligibility criteria	5	Specify the inclusion and exclusion criteria for the review and how studies were grouped for the syntheses.	Pg 4
Information sources	6	Specify all databases, registers, websites, organisations, reference lists and other sources searched or consulted to identify studies. Specify the date when each source was last searched or consulted.	Pg 4
Search strategy	7	Present the full search strategies for all databases, registers and websites, including any filters and limits used.	Supplement 1
Selection process	8	Specify the methods used to decide whether a study met the inclusion criteria of the review, including how many reviewers screened each record and each report retrieved, whether they worked independently, and if applicable, details of automation tools used in the process.	Pg 4-5

© 2022. Zonneveld MH et al. Published online at http://www.aginganddisease.org/EN/10.14336/AD.2022.0818

SUPPLEMENTARY DATA

Data collection process	9	Specify the methods used to collect data from reports, including how many reviewers collected data from each report, whether they worked independently, any processes for obtaining or confirming data from study investigators, and if applicable, details of automation tools used in the process.	Pg 4-5
Data items	10a	List and define all outcomes for which data were sought. Specify whether all results that were compatible with each outcome domain in each study were sought (e.g. for all measures, time points, analyses), and if not, the methods used to decide which results to collect.	Pg 5
	10b	List and define all other variables for which data were sought (e.g. participant and intervention characteristics, funding sources). Describe any assumptions made about any missing or unclear information.	Pg 5
Study risk of bias assessment	11	Specify the methods used to assess risk of bias in the included studies, including details of the tool(s) used, how many reviewers assessed each study and whether they worked independently, and if applicable, details of automation tools used in the process.	Pg 4-5
Effect measures	12	Specify for each outcome the effect measure(s) (e.g. risk ratio, mean difference) used in the synthesis or presentation of results.	Pg 5
Synthesis methods	13a	Describe the processes used to decide which studies were eligible for each synthesis (e.g. tabulating the study intervention characteristics and comparing against the planned groups for each synthesis (item #5)).	Pg 5
	13b	Describe any methods required to prepare the data for presentation or synthesis, such as handling of missing summary statistics, or data conversions.	Pg 5
	13c	Describe any methods used to tabulate or visually display results of individual studies and syntheses.	
	13d	Describe any methods used to synthesize results and provide a rationale for the choice(s). If meta-analysis was performed, describe the model(s), method(s) to identify the presence and extent of statistical heterogeneity, and software package(s) used.	Pg 5-6
	13e	Describe any methods used to explore possible causes of heterogeneity among study results (e.g. subgroup analysis, meta-regression).	Pg 5
	13f	Describe any sensitivity analyses conducted to assess robustness of the synthesized results.	N.A.
Reporting bias assessment	14	Describe any methods used to assess risk of bias due to missing results in a synthesis (arising from reporting biases).	Pg 5
Certainty assessment	15	Describe any methods used to assess certainty (or confidence) in the body of evidence for an outcome.	Pg 5

© 2022. Zonneveld MH et al. Published online at http://www.aginganddisease.org/EN/10.14336/AD.2022.0818
